# The Relationship of Liver Function Tests to Mixed Exposure to Lead and Organic Solvents

**DOI:** 10.1186/2052-4374-25-5

**Published:** 2013-05-21

**Authors:** Won-Joon Chang, Kyu-Tak Joe, Hye-Young Park, Jong-Do Jeong, Duk-Hee Lee

**Affiliations:** 1 Department of Occupational and Environmental Medicine, Dankook University Hospital, Cheonan, South Korea; 2 Graduate School of Public Health, Kyungpook National University, Daegu, South Korea; 3 Department of Occupational and Environmental Medicine, Pohang Sunlin Hospital, Pohang, South Korea; 4 Department of Preventive Medicine and Health Promotion Research Center, School of Medicine, Kyungpook National University, Daegu, South Korea

**Keywords:** Exposure, Lead, Solvents

## Abstract

**Objective:**

This study aims to compare liver function indices (aspartate aminotransferase [AST], alanine aminotransferase [ALT], and gamma glutamyl transferase [GGT]) among males who work with lead, organic solvents, or both lead and organic solvents, under the permissible exposure limit (PEL).

**Methods:**

A total of 593 (out of 2,218) male workers who agreed to share their personal health information for medical research were selected for this study. Those excluded were hepatitis B carriers, individuals exposed to occupational risk factors other than lead and organic solvents, and individuals without liver function results. The 593 were divided into five groups: a lead-exposed group, an organic solvent-exposed group exposed to trichloroethylene (TCE co-exposed solvent group), an organic solvent-exposed group not exposed to trichloroethylene (TCE non-exposed solvent group), a lead and organic solvent-exposed group (mixed exposure group), and a non-exposed group (control group).

We performed a one way-analysis of variance (one way-ANOVA) test to compare the geometric means of liver function indices among the groups, using a general linear model (GLM) to adjust for age, work duration, body mass index (BMI), smoking, and alcohol intake. In addition, we performed a binary logistic regression analysis to compare the odds ratios among groups with an abnormal liver function index, according to a cut-off value.

**Results:**

The ALT and AST of the mixed exposure group were higher than those of the other groups. The GGT of the mixed exposure group was higher than the TCE co-exposed solvent group, but there was no difference among the control group, TCE non-exposed solvent group, lead-exposed group, and mixed exposure group. The same result was evident after adjusting by GLM for age, work duration, BMI, smoking, and alcohol intake, except that ALT from the mixed exposure group showed no difference from the TCE co-exposed solvent group.

When the cut-off values of the AST, ALT, and GGT were 40 IU/L, 42 IU/L, and 63 IU/L, respectively, a logistic regression analysis showed no differences in the odds ratios of those who had an abnormal liver function index among the groups. However, if the cut-off values of the AST, ALT, and GGT were 30 IU/L, 30 IU/L, and 40 IU/L, respectively, the odds ratio of the AST in the mixed exposure group was 4.39 (95% CI 1.86-10.40) times higher than the control.

**Conclusion:**

This study indicates that a mixed exposure to lead and organic solvents is dangerous, even if each single exposure is safe under the permissible exposure limit. Therefore, to ensure occupational health and safety in industry, a continuous efforts to study the effects from exposure to mixed chemicals is needed.

## Introduction

Presently, various organic solvents are being used in the workplace, and numerous toxicities are reported due to occupational organic solvent exposure. In case of occupational organic solvent exposure, the amount of occupational exposure is relatively low due to legal regulations on exposure, personal protective equipment, periodic special health examinations, and workplace environmental assessment. However, there is little known about the toxicity threshold level when organic solvents are combined with other toxins [1]. Thus, a combined chemical toxicity is being overlooked in current safety guidelines.

Organic solvents are very toxic to the nervous system, liver, kidney, and heart [2,3]. Generally, halogenated hydrocarbons and nitrogenated hydrocarbons have greater toxicity than aromatic hydrocarbons or aliphatic hydrocarbons. Therefore, organic solvents with strong toxicity are being replaced with ones with weaker toxicity. However, exposure to a large quantity of weaker hydrocarbons can still cause acute liver necrosis, fatty liver, and hepatorenal syndrome. It was reported that hepatorenal syndrome is induced by habitual glue sniffing in toluene abuse [4].

Past studies on the impact of organic solvents on liver function have mostly dealt with high dose and single exposure cases, such as acute liver disease caused by dimethylforamide (DMF) [5,6], acute hepatitis caused by trichloroethylene (TCE) [7], and the association of liver function and organic solvent exposure [8]. So far, studies on low dose exposure to organic solvents have shown no relationship with liver toxicity [9]. However, liver damage can occur when combined with medication or alcohol [10-12]. Therefore, even if a worker is exposed to low dose organic solvents, liver damage could be increased by additive action or interaction in the case of combined exposure to additional hepatotoxic chemicals.

Early diagnosis of organic solvent toxicity by conventional methods is difficult because the tests lack the proper level of sensitivity. Moreover, hepatotoxicity of organic solvents is affected by many factors such as species differences, liver blood flow, protein binding, point of intracellular binding, genetic factors, different cellular enzymatic degradation, age, nutritional condition, interaction with alcohol, and interaction with use or abuse of drugs [10]. For this reason, Brautbar et al. [10] emphasized that cofactors plays a critical role in toxicity from organic solvents in combined chemical exposure.

Recent studies [13] on polychlorinated biphenyls (PCBs), lead, and mercury revealed that lead is associated with significant dose-dependent alanine aminotransferase (ALT) elevation in subjects whose ALT elevations were not explained by viral hepatitis, hemochromatosis, or alcohol abuse. An animal study by Flora et al. [14] showed that lead-induced oxidative stress was elevated additively according to the intake dose of alcohol.

Currently, exposure to multiple organic solvents is regulated under the assumption that there is no interaction among different organic solvents. Each of the measured concentrations of organic solvents is divided by its permissible exposure limit (PEL), and the sum of these reaults is called 'Estimation of mixture (EM)' which is assessed by comparison with 1 [15].

The mechanism of organic solvent-induced liver injury is known to be increased oxidative stress [16]. Lead is also known to cause liver damage by increasing oxidative stress [17]. Thus, when the liver is exposed to these two chemicals, the liver could be damaged additively by the same mechanism of oxidative stress. In the case of mixed expose to lead and organic solvents, hepatotoxicity overlaps because these two chemicals have the same toxicity mechanism. However, presently, the additive action between lead and organic solvents is not taken into account in assessments of toxicity. Therefore, there is a need to analyze the increased impact on liver damage when workers are exposed to organic solvents under the PEL, which is known to be safe for the majority of workers, but the exposure is mixed with an additional hepatotoxic chemical such as lead.

Therefore, in this study, we will investigate whether hepatotoxicity is increased, when workers are co-exposed to lead and organic solvents under the PEL.

## Materials and methods

### Study population

The sample population was collected from July 1 to December 31, 2009 from special and general health examinations at a hospital in the city of Pohang for 4 companies: one steel company with exposure to lead, one non-ferrous metal company with exposure to organic solvents and lead, one paint manufacturing company with exposure to organic solvents and lead, one painting company with exposure to organic solvents.

**Figure 1:**
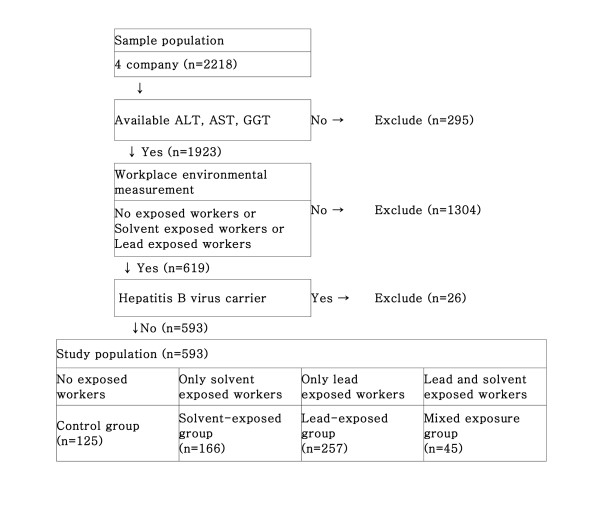
Flow of study participants selection.

2218 adult male workers agreed to share their personal health information for medical research. From this group, those excluded were hepatitis B carriers, individuals exposed to occupational risk factors other than lead and organic solvents, and individuals without liver function test results. In the end, a total of 593 (out of 2,218) male workers were selected for this study (Figure 1).

The lead-exposed group consisted of 257 workers, who, based on the findings of the special health examination, were found to be only exposed to lead. Lead exposure groups were composed of those involved in the melting process at the steel company.

Depending on the degree of toxicity of organic solvents, The organic solvents-exposed group was divided into two groups, 82 workers exposed to organic solvents with TCE (TCE co-exposed solvent group) and 84 workers exposed to organic solvents without TCE (TCE non-exposed solvent group). The workers were exposed to organic solvents including TCE in the washing process at the non-ferrous metal company. They were exposed to organic solvents without TCE in the painting process at the non-ferrous metal company. In the painting company, organic solvents without TCE were used. The processes of the paint manufacturing company were divided into weighing - combining - softening - mixing - packaging. In softening, mixing, and packaging process, organic solvents without TCE were used.

The mixed exposure group consisted of 45 workers who, as a result of the special examination, were found to have a combined exposure to lead and organic solvents. 7 of these workers were exposed to lead and organic solvents without TCE in the combining process of the paint manufacturing company and the rest of the 38 workers were from the non-ferrous metal company's detonator installating process, where they were exposed to lead and organic solvents with TCE. Because of the small number of workers in combination process, they were not divided into TCE and non-TCE mixed exposure groups.

The control group was composed of 125 workers who were not exposed to hazards including organic solvents and lead. In selecting the control group, office workers with no exposure to hazards could have been selected but were excluded in consideration of healthy worker effect.

In each company, the workplace procedure had not changed significantly for the past 30 years, and the companies were considered to be stable with a low employee turnover rate.

## Methods

### Questionnaire

On the day of the special and general health examinations, each worker was asked to complete a questionnaire which was in the practical guideline of the worker's medical examination [18]. The questionnaire included the following items: age, name of company, work process, date of employment, duration of smoking, amount of tobacco smoked per day, duration of drinking, frequency of drinking in a week, and history of illness including hepatitis B. The questionnaire was checked by the nurse who explained that it could be used for research or statistical analysis and a consent form for releasing personal information was signed by the worker.

### Height and weight

Height and weight were measured by an electronic height machine (FA-94H, Fanics, Korea), measured to the nearest 1 cm and 1 kg. The body mass index (BMI) was calculated by dividing the weight by the height in meters squared.

$$\mathrm{BMI}=\frac{\text{mass}\;\left(\mathrm{kg}\right)}{{\left(\text{height}\;\left(m\right)\right)}^{2}}$$

### Liver function test

Fasting was conducted from 10 p.m. the day before the examination. On the day of the examination, 5 ml of blood was drawn from the artery and a liver function test was conducted by an automatic chemical analyzer (Automatic chemical analyzer, Hitachi 7180, Japan).

### Lead concentration in the blood

Lead concentration in blood was measured from 3 ml of whole blood using an automatic absorption spectrometer (Atomic absorption spectrometer-200HT, Varian, Australia).

### Urine hippuric acid and methyl hippuric acid

Urine was collected after 8 hours of work. Urine creatinine was measured by an automatic chemical analyzer (Automatic chemical analyzer, Hitachi 7180, Japan). Urine hippuric acid and urine methyl hippuric acid were measured by high performance liquid chromatography (High performance liquid chromatography, Agilent 1200, U.S.A.) and adjusted by urine creatinine as follows [19].

$$\frac{\text{Concentration}\;\text{of}\;\text{analyte}\;\text{in}\;\text{urine}}{\text{Concentration}\;\text{of}\;\text{createinine}\;\text{in}\;\text{urine}\;}$$

### Selection of exposed hazards and analysis of specimen

#### Selection of hazards

A hazard is any source of potential damage, harm, or adverse health effects on something or someone under certain conditions at work. In the special health examination in the Republic of Korea, the hazards consisted of 177 chemicals and physical materials.

The selection of hazards was conducted through a preliminary survey to understand the flow of the work process. Then, based on material safety data sheets (MSDSs), if the process contained more than 1% of the hazard, it was identified as a hazardous process. Among these processes, temporary work that took less than 24 hours per month or less than 1 hour of work per day was excluded from the hazard selection.

This study enrolled the workers in processes in which they were exposed to hazardous lead or/and organic solvents and those in the processes by which they were not exposed to any hazardous factor according to the workplace environmental assessment for the special health examination.

#### Airborne organic solvent sampling and analysis

The sampling and analysis for airborne lead was performed according to the National Institute for Occupational Safety and Health (NIOSH) Manual of Analytical Methods [20]. By a personal air sampler (Personal air sampler, Gilian Co, USA) using charcoal tube (Coconut shell charcoal tube, SKC, USA), airborne organic solvents were measured near workers' breathing zone for 6–8 hours at a flow rate of 0.2 ℓ/min. The flow rate was calibrated by an electronic flow calibrator (The Gilibrator, Gilian, USA) before and after sampling. For sample preparation, sampling charcoal tubes were broken and the glass wool and foam was discarded. For desorption, 1.0 ㎖ Carbon disulfide was added and allowed to stand for 30 min with occasional agitation. The extracted organic solvents were analyzed by Gas chromatography (Gas chromatography, Shimadzu GC-17A, Japan).

#### EM in airborne organic solvent mixture

According to 'The exposure standards of chemical materials and physical factors' [15], when the body is exposed to two or more contaminants, an additive effect can occur when the contaminants have the same target organ. In this situation, the total effect upon the body equals the sum of the effects from the individual substances. Therefore, estimation of mixture(EM) calculated by the following formula:

EM=C1/T1+C2/T2+⋅⋅⋅⋅⋅⋅⋅⋅Cn/Tn

Where C1, C2, C3… Cn are the measured airborne concentrations of the particular substances 1, 2… n and T1, T2, T3… Tn are the exposure standards for the individual substances.

#### Airborne lead sampling and analysis

The sampling and analysis for airborne lead was performed according to the NIOSH Manual of Analytical Methods [21]. By a personal sampling pump using flexible MCE (Combined Cellulose Ester membrane filter, SKC, USA) filter paper (37 mm, pore size 0.8 μm) in the cassette filter holder, airborne lead was measured near the face of the worker for 6-8hours at a flow rate of 0.7~1.0 ℓ/min. The pump flow rate was calibrated by an electronic flow calibrator (The Gilibrator, Gilian, USA) before and after sampling. The analyte in the MCE filter paper was analyzed using an atomic absorption spectrometer (Atomic absorption spectrometer-200HT, Varian, Australia).

### Statistical analysis

Depending on the exposure to hazardous factors, the study population was divided into the no exposure (control) group, organic solvent-exposed group including TCE (TCE co-exposed group), organic solvent-exposed group not including TCE (TCE non-exposed group), lead-exposed group (lead-exposed group), and the organic solvent- and lead-exposed group (mixed exposure group).

According to tobacco consumption, workers were classified into two groups, smokers and non-smokers. Ex-smokers were classified as smokers. Alcohol intake was calculated as the average of the frequency per week.

From demographic characteristics, the continuous variables were compared by one-way analysis of variance (ANOVA) and independent samples t-test, while smoking, which is a categorical variable, was compared through the chi-squared test.

Liver function indices (AST, ALT, and GGT) showed skewed distribution towards the left; therefore, after log substitution, the geometric means were compared among the groups by one-way ANOVA or a general linear model (GLM).

Recent studies have shown that smoking has an effect on liver function [22-24], it deteriorates viral hepatitis and is a risk factor for non-alcoholic fatty liver [24]. Therefore, factors that have an effect on liver function such as age, body mass index (BMI), work duration, smoking, and alcohol intake were adjusted by a general linear model and the post-hoc test is based on Bonferroni's method.

To compare workers who had abnormal liver function index values (AST, ALT, and GGT) in each group, the odds ratios were calculated according to the cut-off value by binary logistic regression analysis adjusting for age, work duration, body mass index, smoking, and alcohol intake. The cut-off values of liver function index were taken as AST 40 IU/L, ALT 42 IU/L, and GGT 63 IU/L, according to the hospital's values for the special health examination. In addition, to distinguish the differences among the odds ratios by low cut-off values, cut-off values proposed by Lijz et al. [25] of AST 30 IU/L and ALT 30 IU/L were used. There was no discussion of the normal value for GGT, so 40 IU/L was randomly chosen.

The analysis was done by Statistical Package for Social Science Version 18.0 software program (SPSS version 18.0) and a P value < 0.05 was considered statistically significant.

## Results

### General characteristics of the study population

The mean the age of TCE co-exposed organic solvent group was 47.3±6.8 years, which was significantly higher than the age of the other groups. Among the other groups, there was no significant difference. The mean work duration was significantly higher in the TCE co-exposed organic solvent group than the other groups. The other groups did not differ significantly. The body mass index showed no difference among the groups. In case of alcohol intake frequency per week, the mean frequency was significantly lower by 1.31±1.34 per week in the mixed exposure group than the other groups. There were not any significant differences among the other groups. The smoking rate was highest in the TCE non-exposed organic solvent group and lowest in the TCE co-exposed organic solvent group (Table 1).

**Table 1:** Characteristics of the study population unit: mean±SD, number (%)

**Characteristics**		**Control (n=125)**	**Solvent exposure**		**Lead-exposed (n=257)**	**Mixed exposure (n=45)**	**p-value**
			TCE^* ^**co-exposed (n=82)**	**TCE non-exposed (n=84)**			
Age		42.1±9.1^a^	47.3±6.8^b^	39.8±8.0^a^	44.0±10.5^a^	38.8±11.5^a^	<0.000^‡^
Work duration		14.4±11.9^a^	24.5±7.3^b^	14.0±7.5^a^	14.5±9.3^a^	15.1±11.6^a^	<0.000^‡^
BMI^†^		23.9±2.9^a^	22.7±3.5^a^	23.2±3.2^a^	24.2±2.9^a^	24.1±3.8^a^	0.002^‡^
Smoking							
	No	30(24.0)	32(39.0)	14(16.7)	73(28.4)	15(33.3)	0.017^§^
	Yes	95(76.0)	50(61.0)	70(83.3)	184(71.6)	30(66.7)	
Alcohol drink frequency per week							
		2.03±1.44^a^	1.65±1.53^a,b^	1.96±1.54^a^	1.95±1.62^a^	1.31±1.34^b^	0.046^‡^
Blood Lead(μg/dl)		-	-	-	8.33±5.18	6.12±4.30	0.012^∥^
Urine hippuric acid		-	0.57±0.58^a^	0.28±0.36^b^	-	0.37±0.54^b^	0.001^‡^
Urine methyl hippuric acid		-	0.04±0.10^a^	0.14±0.17^b^	-	0.07±0.12^a^	<0.000^‡^

^*^Trichloroethylene, ^†^Body mass index, ^‡^p-values were tested by one-way ANOVA.

^§^p-values were tested by Pearson's Chi-squared test.

^∥^p-value was tested by independent t-test.

^a,b^The same letters indicate a non-significant difference between groups based on Duncan's multiple comparison test.

In case of biological markers, only the workers who were exposed to hazard in the workplace environmental assessment were examined. The blood lead was significantly higher in the lead-exposed group which was 8.33±5.15 ug/dl on average than the combined exposed group which showed 6.12±4.30 ug/dl in average. Urine hippuric acid was found to be significantly higher in the TCE co-exposed organic solvent group 0.57±0.58 than in the other groups, and the urine methyl-hippuric acid was significantly lower in the TCE non-exposed organic solvent group than in the others. The biological markers in the mixed exposure group were not higher than in the other groups (Table 1).

### Workplace environmental assessment

There were 3 companies that used the organic solvents: the non-ferrous metal company, paint manufacturing company and painting company. Among these, the non-ferrous metal company's detonator synthesis and washing processes used organic solvents containing trichloroethylene; other than this, there were no processes that used halogenated or nitrogenated organic solvents.

The exposure of organic solvents was not to a single organic solvent but to combined organic solvent mixtures. The maximum level of EM was 0.5454, which is just half of the PEL (Table 2).

**Table 2:** Organic solvent concentrations in the company unit: ppm

**Organic solvent**	TWA^*^	**Non-ferrous metal company**			**Paint manufacturing company**					**Painting company**	
		**Detonator synthesis**	**Washing**	**Painting**	**Combinating**	**Softening**	**Mixing**	**Mixing**	**Packaging**	**Painting**	**Painting**
		**Mixed exposure**	TCE^†^ co-exposed	**TCE non-exposed**	**Mixed exposure**	**TCE non-exposed**					
n-hexane	50	0.1031	0.1112	trace	-	-	-	-	-	-	0.0835
Ethyl acetate	400	0.2319	0.3808	0.5342	2.8413		-	2.0855	-	-	-
MEK^‡^	200	0.1041	0.1946	0.1656	4.1674	12.7069	0.5546	9.1642	11.2934	-	-
MIK^§^	50	0.6948	0.9361	0.0572	2.5129	8.7895	0.0045	4.4972	2.6295	0.1358	1.4292
Toluene	50	1.6478	2.442	0.7003	5.4129	5.9577	2.1633	8.9979	4.2588	0.9049	12.4106
Acetone	500	-	-	-	-	-	-	-	-	0.2642	9.1653
IPA^∥^	200	-	-	-	-	-	-	-	-	-	9.3673
TCE	50	8.1785	11.658	±	-	-	-	-	-	-	-
n-butyl Acetate	150	-	0.5145	0.6153	-		-	1.2671	-	-	-
Xylene	100	0.3448	0.0388	0.2195	4.0403	18.6873	4.9197	7.8636	10.8343	0.2108	5.1361
Ethyl ether	400	0.0135	-	-	-	-	-	-	-	-	-
EM^¶^	1	0.2178	0.3103	0.0241	0.2269	0.5454	0.0953	0.408	0.3026	0.0234	0.41

^*^Time weighted average, ^†^Trichloroethylene, ^‡^Methyl ethyl ketone, ^§^Methyl isobutyl ketone, ^¡«^Isopropyl alcohol, ^¶^ Estimation of mixture.

The processes giving a combined exposure to lead and organic solvents were the detonator installation process from the non-ferrous metal company and the mixing process from paint manufacturing company. The detonator installation process workers were exposed to organic solvents including TCE and mixing process workers of the paint manufacturing company were exposed to organic solvents without TCE. The EM of organic solvents in mixed exposure group was measured at 0.2178 in the detonator installation process and 0.2269 in the mixing process, which is 1/4 of the legal permissible exposure limit (Table 2).

The processes that involved the lead were the steel company's melting process, the non-ferrous metal company's detonator synthesis process, and the combination process of the paint manufacturing company. The highest concentration of lead was 0.0207 mg/m^3^ which was below the legally permitted 0.05 mg/m^3^ (Table 3).

**Table 3:** **Lead concentrations in the company unit: mg/m**^**3**^

**Company**	**Work process**	**Group**	**Location**						**Maximum**	**Minimum**	TWA^*^
			**1**	**2**	**3**	**4**	**5**	**6**			
Steel company	Steel melting	Lead exposed	0.0149	0.0052	0.0164	0.0207	0.0098	0.0002	0.0207	0.0002	0.05
Non-ferrous metal company	Detonator synthesis	Mixed exposure	0.0045	0.0048	0.0064	0.0049	0.0051	0.0052	0.0064	0.0045	0.05
Paint manufacturing company	Combination	Mixed exposure	0.0025	0.0088	0.0045	0.0024	0.0028	0.0014	0.0088	0.0014	0.05

^*^Time weighted average.

### Liver function indices

Without adjustment for covariates, the AST for the mixed exposure group was found to be at 26.9±1.3 IU/L than in the other groups. The ALT was also higher in the mixed exposure group than the others, at 29.5±1.6 IU/L. The GGT in the mixed exposure group was 39.8±1.5 IU/L. Which was higher than the TCE co-exposed organic solvent group, but other than that, it was the same as in the other groups (Table 4).

**Table 4:** Comparison of geometric means of the AST, ALT, and GGT unit: IU/L

		**Control (n=125)**	**Solvent-exposed**		**Lead-exposed (n=257)**	**Mixed exposure (n=45)**	**p-value**
			**TCE**^**§ **^**co-exposed (n=82)**	**TCE non-exposed (n=84)**			
No adjustment							
	AST^*^	21.9±1.3^a^	22.6±1.4^a^	23.2±1.3^a^	23.4±1.4^a^	26.9±1.3^b^	0.005^¡«^
	ALT^†^	23.4±1.5^a^	22.7±1.6^a^	23.2±1.6^a^	24.5±1.6^a^	29.5±1.6^b^	0.028^¡«^
	GGT^‡^	33.9±1.9^a,b^	30.0±1.7^a^	33.2±1.8^a,b^	38.9±2.0^b^	39.8±1.5^b^	0.009^¡«^
After adjustment							
	AST	22.6±1.0^a^	22.8±1.0^a^	23.8±1.0^a^	23.5±1.0^a^	28.9±1.0^b^	<0.000^¶^
	ALT	23.6±1.0^a^	24.3±1.1^a,b^	23.6±1.1^a^	24.0±1.0^a^	30.0±1.1^b^	0.032^¶^
	GGT	34.6±2.0^a,b^	30.0±1.7^a^	33.2±1.8^a,b^	39.1±2.0^a,b^	39.9±1.6^b^	0.021^¶^

^*^Aspartate aminotransferase, ^†^Alanine aminotransferase, ^‡^Gamma glutamyl transferase, ^§^Trichloroethylene.

^∥^p-values were tested by one-way ANOVA. and the post-hoc test was based on Duncan's method.

^¶^p-values were tested by general linear model adjusted for age, body mass index, work duration, smoking, and alcohol intake by general linear model. The post-hoc test was based on Bonferroni's method.

^a,b^The same letters indicate a non-significant difference between groups.

With adjustment for age, work duration, body mass index, smoking, and alcohol intake, The AST and GGT results were the same as those without adjustment. With adjustment for age, work duration, body mass index, smoking, and alcohol intake, the ALT in the mixed exposure group was shown to be 30.0±1.1 IU/L, which was not significantly different from the TCE co-exposed group at 24.3±1.1 IU/L, but significantly higher than the other groups. The AST in mixed exposure group was higher at 28.9±1.0 IU/L than in the other groups. The GGT in the mixed exposure group was higher at 39.9±1.6 IU/L than in the TCE co-exposed group at 30.0±1.7 IU/L, but other than that, there were no differences among the other groups (Table 4).

### Logistic regression analysis

A logistic regression analysis was conducted to obtain odds ratios of workers with abnormal liver indices exceeding the cut-off value of each group. The results in which the hospital cut-off value was adopted showed no significant difference among the groups (Table 5). However, adopting the cut-off values proposed by Lijz et al. [25], AST 30 IU/L and ALT 30 IU/L, the odds ratio of abnormal AST in the mixed exposure group was significantly higher at 4.39 (95% CI 1.86-10.40) times than the control group. In case of an abnormal ALT, the odds ratio in the mixed exposure group was high at 2.10 (95% CI 0.94-4.46), compared to the control group, but the difference was not significant (Table 5). This indicates that when the cut-off value is set low, as Lijz et al. [25] proposed, the difference between each group's geometric mean of the liver function index is very well illustrated. This also means that the difference among each group's geometric mean of the liver function index is within the margin of the normal value.

**Table 5:** Number of workers with abnormal liver function index values, odds ratios, and 95% confidence intervals according to the cut-off value

			**Number (%)**	OR^*^	**95% CI**^**†**^
The cut-off value of AST^‡^, ALT^§^, and GGT^∥^ were taken to be 40 IU/L, 42 IU/L, and 63 IU/L					
AST	Control		4 (3.2)		
	Solvent-exposed	TCE^¶^ co-exposure	3 (3.7)	1.02	0.21-4.96
	Solvent-exposed	TCE non-exposure	2 (2.4)	0.84	0.15-4.76
	Lead-exposed		21 (8.2)	2.66	0.87-8.17
	Mixed exposure		2 (4.4)	1.46	0.25-8.57
ALT	Control		12 (9.6)		
	Solvent-exposed	TCE co-exposure	8 (9.9)	1.36	0.49-3.78
	Solvent-exposed	TCE non-exposure	10 (11.9)	1.36	0.54-3.45
	Lead-exposed		35 (13.6)	1.49	0.72-3.06
	Mixed exposure		7 (15.6)	1.55	0.23-4.54
GGT	Control		24 (19.2)		
	Solvent-exposed	TCE co-exposure	8 (9.9)	0.52	0.20-1.34
	Solvent-exposed	TCE non-exposure	8 (9.5)	0.50	0.20-1.22
	Lead-exposed		63 (24.5)	1.32	0.75-2.32
	Mixed exposure		7 (15.6)	0.97	0.35-2.66
The cut-off value of AST, ALT, and GGT were taken to be 30 IU/L, 30 IU/L, and 40 IU/L					
AST	Control		15 (12.0)		
	Solvent-exposed	TCE co-exposure	8 (9.9)	0.80	0.31-2.09
	Solvent-exposed	TCE non-exposure	9 (10.7)	1.00	0.31-2.45
	Lead-exposed		44 (17.1)	1.42	0.75-2.70
	Mixed exposure		15 (33.3)	4.39	1.86-10.40
ALT	Control		41 (32.8)		
	Solvent-exposed	TCE co-exposure	18 (22.2)	0.94	0.45-1.94
	Solvent-exposed	TCE non-exposure	22 (26.2)	0.81	0.41-1.58
	Lead-exposed		89 (34.6)	1.04	0.64-1.71
	Mixed exposure		19 (42.2)	1.73	0.78-3.82
GGT	Control		41 (32.8)		
	Solvent-exposed	TCE co-exposure	24 (29.6)	1.27	0.63-2.56
	Solvent-exposed	TCE non-exposure	25 (29.8)	1.13	0.59-2.18
	Lead-exposed		104 (40.5)	1.35	0.83-2.19
	Mixed exposure		18 (40.0)	2.10	0.94-4.68

^*^Adjusted for age, work duration, body mass index, smoking, alcohol by binary logistic regression model.

^†^Confidence interval, ^‡^Aspartate aminotransferase, ^§^Alanine aminotransferase, ^∥^Gamma glutamyl transferase, ^¶^Trichloroethylene.

## Discussion

Liver disease initially appears in the form of fatty liver. As it progresses, it deteriorates into hepatitis, liver cirrhosis, and liver cancer. Solvents may damage liver cells and liver transaminases may be used to monitor liver damage. Liver disease cannot be confirmed with a blood chemistry test, but clinically, ALT is most commonly used as a biomarker of liver damage. In the liver function test, ALT, AST, and GGT can be elevated by other organ damage than liver [26]. Thus, in order to increase the sensitivity, different biochemical parameters of liver function should be measured [27], such as alkaline phosphatase, albumin, and bilirubin.

ALT is an enzyme involved in the transfer of an amino group from alanine and present in the cytoplasm. ALT is found in various tissues but is most commonly associated with the liver. Therefore, ALT is a good biomarker of hepatocelluar injury [28]. AST is an enzyme involved in the transfer of an amino group from aspartate. More than 80% of AST is present in the mitochondria and the remaining 20% of AST is present in the cytoplasm. Thus, cytosolic AST (cAST) promptly appears in the blood from an injured cell but mitochondrial AST (mAST) remains in the core regions of an injured cell. Thus, mAST in the blood reflects the more severe cell damage or necrosis [29]. In case of alcoholic hepatitis, mainly the mitochondria are damaged. Thus, AST increases more than ALT [28].

Unlike membrane-bound enzyme, cytosolic enzyme does not leak into blood. Healthy plasma membranes should be impermeable to macromolecules such as enzymes. It is generally accepted that increased cytosolic enzyme in the blood occurs secondary to cell membrane damage or cell necrosis [30]. Therefore, increased AST and ALT are biomarkers of hepatic injury rather than hepatic dysfunction [28].

GGT is a membrane-bound enzyme found in the kidneys and liver. Renal GGT is excreted into the urine, not the blood. Hepatic GGT has direct access to the blood. Hence, most of the serum GGT activity in the blood is from the liver. GGT is released into blood by cellular injury, cholestasis, or overproduction which is induced by medication [30]. Elevated GGT alone may be found in chronic alcoholics. Hence, the change in GGT can be used to verify the latest intake of alcohol in chronic alcoholics. Recently, Lee et al. [31] suggested that GGT can be used not only as a liver function index and biomarker of alcohol intake but also as a sensitive biomarker of oxidative stress.

This study compared and analyzed the geometric mean of liver function indices of single exposure and the mixed exposure in male workers who were exposed to lead and/or organic solvents under the legally permissible exposure limit. Today, the workers are exposed to organic solvents within the legally permissible exposure limit base on workplace environmental assessment and special examinations. However, despite this low dose exposure levels within the PEL, there are many reports of unexplained liver diseases for cause other than viral hepatitis, alcoholic hepatitis, obesity, and hemochromatosis. It is presumed that these unexplainable liver disease cases are caused by environmental pollutants, drug use, and industrial chemicals.

In National Health and Nutrition Examination Survey (NHANES) 1988–1994, Clark et al. [32] reported that the unexplained ALT elevation was 5.4%. Cave et al. [13] reported an unexplained ALT elevation of 10.6% in the NHANES 2003–2004 survey and this was associated with high-level chemical exposure. Cave et al. [13] suggested that an unexplained ALT elevation had a dose-dependent relationship with using of the chlorinated biphenyls (PCBs), lead, mercury, etc., even from low-dose single exposure.

Vaziri et al. [33] suggests that lead is associated with mild up-regulation of superoxide-generating enzyme. Farmand et al. [17] suggests that lead induces reactive oxygen species, which deplete antioxidants and destabilize cell membranes by attacking the fatty acids of the cell membrane. Lee et al. [34] proposes that lead-induced antioxidant depletion increases the oxidative stress marker, GGT.

ALT and GGT are useful biomarkers before liver damage is irreversible [35,36]. Organic solvent-induced liver disease can be prevented from becoming irreversible liver disease by removal of solvent exposure, if detected at an early stage [37]. Hence, ALT and GGT in organic solvent-exposed workers are useful biomarkers that can detect liver damage early and allow for follow-up.

The study of Ukai et al. [9] on exposure to organic solvents within the PEL did not show any effect on the liver. The study of Nasterlack et al. [38] on exposure within the PEL to organic solvents did not show any elevation of AST, ALT, or GGT. However, Goji et al. [11] state that painting work rarely affects liver function when the worker is exposed to organic solvents. However, in the case of workers who drink alcohol, exposure to organic solvents cause the liver function to deteriorate. Thus, exposure to organic solvents combined with other hazard factors may show an effect on the liver, even if within the PEL. Brautbar et al. [10] reported that hepatotoxicity of organic solvents is changed by various factors and the cofactors play a major role.

In the study on “Dose-dependent effects of ethanol on lead-induced oxidative stress in rats” by Flora et al. [14], greater decrease in antioxidant materials such as GSH and greater increase in oxidant materials such as reactive oxygen species was reported in case of co-exposure to lead and ethanol rather than a single chemical exposure. This result suggests that lead induced-oxidative stress escalates the oxidative stress induced by alcohol.

The main pathogenic mechanisms responsible for functional and organic damage caused by organic solvents can be classified into four types: 1. inflammation, 2. dysfunction of cytochrome P450, 3. mitochondrial dysfunction, and 4. oxidative stress [16]. These 4 mechanisms result in increasing oxidative stress. Thus, the oxidative damage caused by free radicals is thought to be a basic mechanism underlying hepatotoxicity by organic solvents [16].

Already more than 50 years ago, three basic types of action for combinations of chemicals were defined [39,40] as follow: 1. similar action (additive action), 2. dissimilar action (independent action), 3. interactions (classified into antagonism, inhibition, synergism, and potentiation). If the target organ is different, independent action occurs. However, when the target organ is the same, the toxicity shows different results based on the mode of action. In general, chemicals with common modes of action act additively and chemicals with different modes of action show interaction [41]. Interaction usually occurs at medium or high dose levels. At low exposure levels, such as in this study, interactions either do not occur or are toxicologically insignificant [41].

In view of the almost infinite number of possible combinations of chemicals to which humans are exposed and the fact that the mode of action for each material is unknown, a guideline to evaluate combined materials is needed. Based upon these facts, a decision tree for evaluating the risk of chemical mixtures was proposed by the European Union (EU) in 2011. In this report, when the mode of action is uncertain, then, it is recommended to assume additive action [41].

Presently, the combined exposure of organic solvents is evaluated additively under the assumption that there are no interactions among them. However, in the workplace, combined materials other than organic solvents are evaluated as single chemicals independently without considering their interaction or additive action.

Recently, in several regions, including Europe and the United States, an effort have been made to develop a web-based computer tool which allows the user to determine whether there is potential additive action or interaction among components of a mixture [42,43]. These efforts are at the level of risk assessment, not for practical use.

Many studies on the mode of action between organic solvents show additive action or a synergistic effect [44]. However, that was not considered in this study. Instead, this study classified organic solvents into two groups, one including the highly toxic halogenated organic solvent, TCE, and the other one without TCE.

This study looked into the effects of lead and organic solvents. The results reveal that the liver function index was more elevated in the mixed exposure group than in the single chemical exposed groups. It can thus be assumed that the toxicity of lead and organic solvents to the liver is additive.

There are a number of studies on combined exposure to chemicals such as endocrine disruptors, heavy metals, and organic solvents [45-47]. However, no studies evaluated the combined exposure of heavy metals and organic solvents, such as the combined exposure to lead and organic solvents. Hence, the results of this study could not be compared to previous research. However, in this study, if the mode of action is the same, that is, oxidative stress and in the same target organ, the liver, then it is reasonable to evaluate the exposure limit as additive.

Normal values are defined as the mean of the distribution ±2 standard deviations of the 'normal' population. Therefore, by definition, 2.5% of healthy people can have a high value. Moreover, most liver diseases lack symptoms or have non-specific symptoms. If a liver function test is normal, it does not ensure that the patient is free of liver disease. Thus, the normal population who are predicted to be healthy may include some with mild liver disease [48,49].

Moreover, AST and ALT are enzymes that exist in cells that appear in the blood in case of cell damage or necrosis. Therefore, from the perspective of the meaning of AST and ALT in liver function test, the term, 'function' is incorrect [28] because they are biomarkers that determine the level of 'damage' to the cells.

According to a recent prospective cohort study by Kim et al. [50], the mortality rate from liver disease rises in accordance with the elevation of AST and ALT even though the AST and ALT are within normal range. Yuen et al. [51] reported through a follow up observation of chronic hepatitis B patients that patients with an ALT level 0.5-1 times the upper limit of normal (ULN) had an increased risk for the development of complications compared with patients with an ALT level <0.5 times the ULN. Lijz et al. [25] suggest that the cut-off value of ALT should be lowered from 40 IU/L to 30 IU/L because the revised cut-off value of ALT can better discriminate between HBeAg(-) chronic active and inactive patients. Many studies [49,52,53] have mentioned the same suggestion to lower the cut-off value. For this reason, the present cut-off value could underestimate the prevalence rate of liver disease, and, as stated in this study, the gap within the normal range should not be ignored.

This study demonstrates very well the gap between the geometric means of AST and ALT but based on logistic regression analysis by the present cut-off value, there were no difference in the odds ratios among the groups. However, by the revised cut-off value, the odds ratio of AST in the mixed exposure group was significantly increased by 4.39 times (95% confidence interval, 1.86-10.40) compared with the control group. Furthermore, the odds ratios of ALT and GGT were increased but not significantly.

There could be three reasons for this. First, a worker with liver disease who had an abnormal liver function test could have gotten treatment; hence, the levels returned to normal. Second, task-switching could exclude a worker who had an abnormal liver function test from the mixed exposure group. Three, the increased range of liver function test in mixed exposure group could be insufficient to exceed the cut-off value, so, it could be within normal range

In case of the third reason, the current normal range of the liver function test cannot distinguish the toxic effect of chemical mixtures under the current PEL. Thus, hepatotoxicity of chemical mixtures is likely to be overlooked clinically. This can be remedy by lowering the cut-off value resulting in increased odds ratios. Therefore, in accordance with the many studies on cut-off values for liver function tests, a revision of the cut-off value is required to detect subclinical disease.

The significance of this study is primarily that it is a study of combined chemical exposure under the PEL. Exposure under the PEL means that it is not harmful to most workers. However, in the case of mixed chemical exposure to the same target organ, additive action and interaction should be considered. In addition, even if exposures are under the PEL, it can be harmful to workers. Secondly, the gap within the normal range should not be ignored.

However, there are many limitations to interpreting this study. First, this is a cross-sectional study. Therefore, the causality can only be an assumption. Hence, verification through additional studies is needed. Second, the classification of the exposure group is supposed to have an accurate exposure evaluation according to an individual biomarker. However, due to the limitations of the data, the exposure groups were classified by hazard factors selected by workplace environmental assessment for special health examination. A well planned study using an individual biomarker will be needed in the near future. Third, this study could not reflect the effects of hepatitis A, hepatitis C, *Clonorchis sinensis* infection, drug use, or herbal medications.

However, despite the limitations mentioned above, this study suggests that the organic solvents exposure mixed with other hazard could cause an elevation of liver function index under the PEL. The elevated liver function index could be within the normal range. Thus, the eleation of liver function can be overlooked. So it is worthy of consideration that, when organic solvent exposed with other chemicals, the liver function index can be increased, althoug the increased value is within the 'normal' range. For this reason, a revision of the PEL is needed to protect the health of workers who are exposed to combination for chemicals. Moreover, the meaning of the liver function test and its normal range should be taken into consideration.

## Competing interests

The authors declare that they have no competing interests.

## Authors’ contributions

D.H. Lee conceived and designed the study. W.J. Chang and K.T. Joe were involved in writing the manuscript. H.Y. Park Performed the data collection. J.D. Jeong performed the statistical analysis, the interpretation of data. All authors read and approved the final manuscript.
